# Direct cost of pars plana vitrectomy for the treatment of macular hole, epiretinal membrane and vitreomacular traction: a bottom-up approach

**DOI:** 10.1007/s10198-015-0741-6

**Published:** 2015-11-24

**Authors:** Elena Nicod, Timothy L. Jackson, Federico Grimaccia, Aris Angelis, Marc Costen, Richard Haynes, Edward Hughes, Edward Pringle, Hadi Zambarakji, Panos Kanavos

**Affiliations:** 1 LSE Health and Social Care, London School of Economics and Political Science, Houghton Street, London, WC2A 2AE UK; 2 Department of Ophthalmology, School of Medicine, King’s College London, London, UK; 3 Department of Ophthalmology, Hull and East Yorkshire Hospital, Yorkshire, UK; 4 Vitreoretinal Unit, Bristol Eye Hospital, Bristol, UK; 5 Vitreoretinal Unit, Sussex Eye Hospital, Brighton, UK; 6 King’s College Hospital, London, UK; 7 Department of Ophthalmology, Barts Health, Whipps Cross Hospital, London, UK

**Keywords:** Cost, Macular hole, Epiretinal membrane, Vitreomacular traction, Pars plana vitrectomy, I1 Health, I19 Other

## Abstract

**Purpose:**

The direct cost to the National Health Service (NHS) in England of pars plana vitrectomy (PPV) is unknown since a bottom-up costing exercise has not been undertaken. Healthcare resource group (HRG) costing relies on a top-down approach. We aimed to quantify the direct cost of intermediate complexity PPV.

**Methods:**

Five NHS vitreoretinal units prospectively recorded all consumables, equipment and staff salaries during PPV undertaken for vitreomacular traction, epiretinal membrane and macular hole. Out-of-surgery costs between admission and discharge were estimated using a representative accounting method.

**Results:**

The average patient time in theatre for 57 PPVs was 72 min. The average in-surgery cost for staff was £297, consumables £619, and equipment £82 (total £997). The average out-of-surgery costs were £260, including nursing and medical staff, other consumables, eye drops and hospitalisation. The total cost was therefore £1634, including 30 % overheads. This cost estimate was an under-estimate because it did not include out-of-theatre consumables or equipment. The average reimbursed HRG tariff was £1701.

**Conclusions:**

The cost of undertaking PPV of intermediate complexity is likely to be higher than the reimbursed tariff, except for hospitals with high throughput, where amortisation costs benefit from economies of scale. Although this research was set in England, the methodology may provide a useful template for other countries.

## Introduction

The English National Health Service (NHS) is a government-funded body responsible for delivering healthcare to England’s public. Government funding is channelled through Clinical Commissioning Groups (CCG). CCGs are responsible for ensuring that services provided in their local area meet patients’ needs, NHS standards and costs, by commissioning NHS hospitals, private sector providers, charities and social enterprises. Introduced in 2003, Payment by Results (PbR) underpins healthcare payments for most hospital care within England’s NHS. PbR sets “tariffs” for a range of interventions. This in turn is defined by Healthcare Resource Groups (HRGs) codes, which are used to classify diagnoses (ICD-10 code) and interventions (OCPS-4). The tariff covers all costs incurred from admission to discharge of the patient. Tariffs are calculated based on national average of costs incurred in the last 3 years within the mandatory reference cost collection system, adjusted for inflation, efficiency (since 2010–2011), and the Market Force Factor (MFF). The HRG4 tariffs, implemented in 2009–2010, also account for co-morbidities, complications, age and length of stay [1].

The objective of PbR was to incentivise improved performance through greater patient choice—payments would follow patients to whichever hospital they chose to attend [2]. This system, however, is not without its challenges. Indeed, the accuracy of the costing data underpinning the tariffs remains poor for certain providers or individual unit costs. Progress has been seen since, but a number of issues remain unresolved [3]. Issues around HRG coding have also resulted in an underpayment of £60 million pounds for acute care and in under and over-payments of between £600 and £700 million for admitted patient care in 2011/2012. Improvements have been seen since the implementation of the PbR assurance framework in recent years, but the value of the errors and the variability amongst providers remain high.

This study seeks to empirically test whether the reimbursed HRG tariff for an ophthalmic surgical procedure called pars plana vitrectomy (PPV) is close to parity with the real costs incurred in NHS hospitals. PPV is the most commonly performed vitreoretinal operation, comprising 71 % of all vitreoretinal procedures in the UK [4]. It is undertaken for a range of indications, but the most common are retinal detachment, macular hole (MH), epiretinal membrane (ERM), and diabetic eye disease [4].

Several studies have attempted to estimate the cost of PPV for a range of conditions [5–13], but only two studies, both from Germany, undertook a bottom-up costing approach [14, 15]. They concluded that the reimbursement for inpatient PPV does not cover the more complex procedures. Most studies estimated costs based on coverage tariffs, that is, the costs were based on what was reimbursed (Table 1).

**Table 1:** Studies from the literature review that assessed the cost of pars plana vitrectomy. *CEA* Cost-effectiveness analysis, *ERM* epiretinal membrane, *PICU* paediatric intensive care unit, *PPV* pars plana vitrectomy, *PVR* proliferative vitreoretinopathy, *RD* retinal detachment

Author	Year	Country	Indication for PPV	Type of study	Type of costs collected	Cost categories	Total cost (£)	Total cost (original currency)^a^
Wisniewski	1997	United States	Endophthalmitis	Top down	Hospital costs	Inpatient treatment costs, usage of intravenous antibiotics	£8280 (IV antibiotics)	US $13,000
							£5796 (no IV antibiotics)	US $9100
Huang	2001	United States	Not specified	Top down	Reimbursement	General and local anaesthesia (operating room, post-anaesthesia care unit, pharmacy and anaesthesia)	£4571 (general anaesthesia)	US $7177
							£3483 (local anaesthesia)	US $5469
Brown	2002	United States	RD with severe PVR	CEA	Reimbursement (Medicare and Medicaid)	Initial office consultation, repair RD, postoperative medications, institutional provider costs, anaesthesia	£3339	US $5243
Sulkes	2002	United States	Endophthalmitis	Top down	Reimbursement (Medicare)	Facility and physician costs	£2362 (inpatient)	US $3708
							£1601 (outpatient)	US $2514
Busbee	2003	United States	Complications of cataract surgery (endophthalmitis, RD)	CEA	Reimbursement (Medicare)	Initial office consultation, PPV, vitreous drug injection, inpatient facility fee, postoperative medications, anaesthesia fees	£3016 (endophthalmitis)	US $4735
							£3304 (RD)	US $5118
Smiddy	2007	United States	Membrane peel, RD	Top down	Reimbursement (Medicare)	Professional, technical and pharmaceutical costs	£2343 (membrane peel)	US $3678
							£2304 (RD)	US $3618
Framme	2007	Germany	Vitreoretinal diseases with and without cataract	Bottom up	Cost collection	Fixed surgical costs, variable costs (personnel, material), hospitalisation and adjunct interventions	£2274	€2661
							£2542 (cataract)	€2974
Framme	2008	Germany	Penetrating eye injuries	Bottom up	Cost collection	Fixed surgical costs, variable costs (personnel, material), hospitalisation and adjunct interventions	£3172	€3712
Lee	2008	United States	Diabetic retinopathy	Top down	Reimbursement (direct cost of procedure, 30-day follow-up costs)	Direct costs include hospitalisation, emergency, outpatient, other medical costs (ancillary services, independent lab, home health)	£4357 (direct cost of PPV)	US $6841
						30-day follow-up costs include hospitalisation, emergency, outpatient, other (productivity losses)	£1985 (cost of 30-day follow-up)	US $3117
Ho	2008	United States	Primary RD	Top down	Reimbursement (hospital charges)	Hospitalisation	£941	US $1478
Gupta	2008	United States	ERM	CEA	Reimbursement (Medicare)	Initial office consultation, ERM surgery, PPV, ambulatory surgical centre fee, anaesthesia, postoperative medications	£2176	US $3417
Kamholz	2009	United States	Retinopathy of prematurity	CEA	Hospital costs	Ophthalmology fee, anaesthesia professional fee, operating room, anaesthesia, PICU observation, general medicine admission, PICU admission	£6612	US $10,381

^a^Original currencies were converted into British pounds (£) using the current exchange rates US $1.57 = £1 and €1.17 = £1

The indications for PPV in these costing studies included diabetic retinopathy [10], retinal detachment [5, 6, 8, 11], eye injuries [15], ERM [7, 11], retinopathy of prematurity [9], and endophthalmitis [6, 12, 13]. A number of cost-effectiveness analyses (CEA) collected reimbursement cost data in the US, and used these figures to populate CEA models [5–7, 9]. The estimated cost of PPV ranged between £1601 (US $2500) for an outpatient with endophthalmitis in Florida (US) [12], to £8280 (US $13,000) for a PPV with intravenous antibiotics in Pennsylvania (US) [13]. The cost of hospitalisation varied between £884 and £1867 ($1388 and €2184) [10, 15], with an average duration of 1 week in those requiring admission [8, 14, 15], but these studies were conducted up to 7 years ago, or included severe eye diseases such as penetrating eye injury, and may not be relevant to more representative cases in 2014–2015. Some studies included the cost of adjunct interventions associated with PPV such as encircling band, perfluorocarbon liquid, indocyanine green used as a vital stain, tissue plasminogen activator used to dissolve submacular hemorrhage, intravitreal gas or silicone oil tamponade, or cataract surgery and as such comprised a heterogeneous mix of cases. In one German study, the additional cost for these adjunct interventions was estimated between £44 and £214 (€51–250) [14]. Another German study reported that 90 % of PPVs included one adjunct intervention, and 50 % included two [15]. One US study examined hospital costs (including operating room, post-anaesthesia care unit, pharmacy and anaesthesia) for patients undergoing PPV with membrane peel with either local or general anaesthesia, which were found to be £3483 (US $5649) and £4571 (US $7177), respectively [16]. In addition to the cost of surgery, two US studies reported the cost of a 30-day follow-up period together with costs of care for the 1st year [10, 11]. All these studies were performed outside the UK and used coverage rather than actual costs, based on a top-down costing approach.

The objective of this study was to estimate the actual direct cost of undertaking PPV of intermediate complexity in a NHS setting, using a bottom-up approach, and compare it to the actual cost reimbursed under the NHS PbR system. Differences in estimates suggest that inefficiencies in the healthcare system exist, including in the incentives implemented [17]. The advantage of using a bottom-up rather than a top-down costing approach is that it accounts for differences in resource use that varies over time and between individuals, which in turn may explain the cost items or drivers contributing to these differences in estimates (across hospitals and with the HRG estimates) [17]. This is particularly important for heterogeneous interventions such as for PPV [15]. Three indications were selected as being representative of a typical intermediate complexity PPV: MH, ERM, and vitreomacular traction (VMT).

## Materials and methods

Five representative, geographically spread, teaching and non-teaching NHS vitreoretinal units were included in the study.

The study selected commonly performed PPV interventions of similar, intermediate complexity (MH, ERM, VMT) in order to ensure a homogeneous sample [4]. Cases that required cataract surgery as part of the PPV were not excluded, to ensure the samples were representative as studies indicate that cataract surgery is undertaken in 27–41 % of cases [4, 18]. Research ethics committee review was not required according to UK guidance, as the study was considered to be either a service evaluation or an economic audit [19].

The direct cost of PPV was estimated based on two sets of data: in-theatre costs and out-of-theatre costs. The former was estimated based on all real costs incurred during surgery, and the latter was estimated using the range of identifiable costs recorded within the accounting system in a representative hospital for a cohort of 31 patients with similar conditions and comprising costs incurred out-of-theatre between admission and discharge of the patient (hereafter referred to as the cohort data), as well as an estimation of nursing and medical staff time before and after surgery. The out-of-theatre costs included a fixed rate for the contribution of different staff (pharmacists and other allied health care professional) as well as clinical, scientific and diagnostic services (including imaging and other diagnostic examinations). Out-of-theatre medical and nursing time were estimated and validated by all partners. This out-of-theatre costing did not consider consumables used outside of the operating theatre with the exception of eye drops, nor did it include other clinical and non-clinical supplies (e.g. information leaflets) or equipment (e.g. recovery equipment, cardiorespiratory monitor).

For the in-theatre data collection, standard data collection templates were created then customised to each site. A site-initiation visit was conducted by two research associates (F.G., A.A.) to perform a general inspection of the operating environment, to provide staff training in data collection, and to record all capital equipment used for performing PPV.

All consecutive NHS PPVs under the care of the named consultant clinical investigator were recorded in a surgical log. The surgical log recorded the surgical elements, date and indication for PPV. The log was completed to provide an estimate of the proportion of PPVs undertaken for the reference indications. The study period began at each site following site initiation, on the day the first surgical list included a case of MH, ERM or VMT. Sites continued to collect data from consecutive PPVs until the surgical log included at least 10 cases of either MH, ERM, or VMT, and also until there was a minimum of 30 PPVs (for any indication). Data collection ran from March to September 2012. No patient details were recorded other than age and indication for surgery. An ophthalmologist or ophthalmic theatre nurse completed the surgical log at the time of surgery. The investigators and hospital managers were advised that any cost data they provide would be anonymised by the research team.

Resource utilisation and cost data were collected on the customised source documents for all PPVs undertaken for the reference conditions during the study period (Table 2). The resource utilisation template, completed by either the ophthalmologist or ophthalmic theatre nurse, collected information on the length of surgery, the theatre and anaesthetic staff involved in surgery, and the consumables used during surgery.

**Table 2:** Data source and cost calculations

In-theatre bottom-up			
	Staff	Consumables	Equipment
Resource use	Resource utilisation log: length of surgery in minutes for all staff involved	Resource utilisation log: list of all consumables used for each surgical intervention	Equipment log: all equipment routinely used for PPV
Cost estimate	NHS salary bands: hourly rate calculated using median values (including employer National Insurance and pension contribution, and high cost area supplements)	Cost log: purchasing price to hospital, accounting for rebates. Any undisclosed costs (8.5 % of items for Hospital 2) were estimated based on the average cost across other hospitals for the same items, when available	Equipment log: cost per PPV estimated using purchasing price (including rebates), yearly maintenance costs, amortisation period and number of operations per year. When data were unavailable, cost estimated at average across other hospitals

Out-of theatre cohort accounting data			
	Staff	Other costs	Overnight stay
Resource use	Per patient perioperative staff time was 7 min for ophthalmologists, 4 min for anaesthetists, 15 min for pre-operative nursing (25 min if general anaesthetic), 15 min post-operative nursing (30 min if general anaesthetic plus 20 min for recovery nurse)	Cohort data: all additional identifiable out-of-surgery costs. Information about the eye drops used before and after surgery were collected from each site	Surgical log: proportion of patients with overnight stay, estimated for each hospital
Cost estimate	NHS salary bands: same as for in-theatre	Cohort data: identifiable costs relating to pharmacist staff costs (fixed cost), merged with diagnostic and laboratory tests (variable cost). The hospital purchasing price was used to estimate the cost of eye drops	NHS HRG reimbursement tariffs for overnight stays

Other			
Overheads: Estimated at 30 % of total costs based on Healthcare Financial Management Association’s (HfMA) clinical costing guidelines			

Costing data were divided into six categories: staff; consumables; equipment; overnight stay; overheads; and other costs.

In-theatre staff costs were calculated based on the number of minutes spent by each staff member in theatre, their position and the median midpoint salary for that band [20]. Hourly rates were based on a 220-day working year and 7.5 h working day, resulting in a total of 1650 h per year. A 10 % national insurance contribution (NIC) and 14 % NHS Scheme Pension employer contribution were added to base salaries, together with the high cost area supplement for inner and outer London [20]. For out-of-theatre nursing staff, we added 15 min pre-operative time for local anaesthetic patients (25 for general anaesthetic patients), and 15 min post-operative time (30 for general anaesthetic patients, with an additional 20 min for the recovery nurse). For each ophthalmologist, we added 7 min for each patient to allow for the preoperative ward round, the time needed to change into surgical scrubs, liaising with nursing and administrative staff, and unforeseen and miscellaneous delays, and patients who failed to attend theatre or were cancelled on the day. For anaesthetic staff we allowed 4 min per patient.

Consumable costs were based on the purchasing price to the hospital, less any rebates. Undisclosed costs were estimated based on the average cost of similar items across other sites. Under “Other costs”, out-of-theatre costs relating to pharmacist staff costs (fixed rate) and diagnostics and laboratory tests (variable rate) were extracted from the cohort data and merged into one cost item, estimated at £94.10. Equipment costs were calculated per PPV based on equipment purchasing price (less rebates), yearly maintenance costs, amortisation period (in years), and number of PPVs performed per year. An allocation was made for each piece of capital equipment, based on the percentage time used for PPV. For example, if an operating microscope was used half the time for PPV and half for other operations, it would have a 50 % cost allocation. When patients stayed overnight, the HRG reimbursement tariff for an overnight stay (£266 per night) was estimated pro rata on the average number of days in hospital, per patient, for each hospital [21].

Overheads were estimated at 30 % of all direct costs based on published NHS guidelines [22]. These account for all costs related to the general management of the hospital, such as costs related to facilities, electricity, general cleaning, management, or finance and legal staff, and which are not driven by the level of patient activity.

The direct cost was compared to the actual amount reimbursed to the hospital for each patient. The reimbursement tariff is determined by published HRG intervention codes [1], under the PbR system. The codes used to classify MH, ERM, VMT and other retinal diagnoses such as age-related macular degeneration or diabetic macular oedema are the same, and are categorised by the level of complexity of the case, from high to low (BZ20Z, BZ21Z, BZ22Z, BZ23Z), ranging between £1823 and £504. These differ from NHS Reference Costs used as a benchmark to calculate the PbR tariffs (£402–£2707).

Consultants completed a questionnaire detailing the advice they provided to patients in terms of time off work, and the time required for head posturing following MH surgery.

## Results

A total of 151 PPVs were recorded during the evaluation, of which 57 (37.7 %) were for the reference conditions. Of the 57 cases, 24 had MH (42.1 %), 22 ERM (38.6 %), five VMT (8.8 %), and six had two or more of the reference conditions (10.5 %). Cataract surgery was performed in 25 cases (43.9 %), and 19 (33.3 %) had an overnight stay. The mean patient age was 72 years (range 63–89) for MH, 71 (46–93) for ERM and 74 (45–82) for VMT.

The average time spent in-theatre was 72 min (range 58–83), and the average number of theatre health professionals involved was 6.5 (range 5–8). This comprised one or two surgeons (such as consultant and fellow), one anaesthetist (such as consultant or associate specialist), one anaesthetic assistant, two circulating nurses, and one scrub nurse. In four of the five sites, all staff allocated 100 % of their time to the PPV, the fifth site allocated between 75 and 100 % of total staff time as they may have been assisting outside of the reference theatre. The average number of consumable items used in-theatre per PPV was 62 (range 35–110), including items used to perform cataract surgery and anaesthesia. The standard equipment used to perform PPV in all sites (and the allocation made to PPV surgery) included a vitrectomy machine (93 %), endolaser (100 %), cryotherapy device (100 %), BIOM lens system and microscope inverter (100 %), and operating microscope (58 %).

The average in-theatre staff cost was £296.90 (range £229.20–£376.70), and the average in-theatre consumables cost was £618.60 (range £509.65–£715.50), including cost estimates for undisclosed costs. The average cost of each equipment item across the four sites was £87,000 for the vitrectomy machine (range £54,000–£120,000), £105,881 for the operating microscope (range £90,284–£126,000), £25,800 for the endolaser (range £10,800–£40,800), £32,654 for the BIOM (range £29,751–£38,210), and £8664 for the cryotherapy machine (range £3613–£13,738). The mean equipment cost per PPV, dividing the yearly cost by the number of PPVs performed per year, was £81.75 (range £47.80–£150.30), with average yearly maintenance costs of £1822.80 (range £880.00–£2555.60), average amortisation period of 8.9 years (range 7.0–10.5), and average annual number of PPVs per machine of 478 (range 225–750).

The estimated average in-theatre cost of PPV was £997.20 (range £825.45–£1166.10), comprising £296.90 for staff, £618.60 for consumables, and £81.70 for capital surgical equipment.

Out-of-theatre costs were estimated as £70.65 (range £55.10–£91.20) for nursing and medical staff costs, £86.90 (range £0.00–£177.35) for hospitalisation and £102.40 (range £99.25–£109.33) for other costs, including eye drops (Table 3).

**Table 3:** Average estimated cost of PPV for ERM, VMT and MH in 5 UK Hospitals. *ERM* Epiretinal membrane, *MH* macular hole, *PPV* pars plana vitrectomy, *VMT* vitreomacular traction

	Staff (in-theatre and out-of-theatre)	Consumables	Equipment	Other costs (including eye drops)	Overnight stay	Total direct cost	Overhead (30 %)	Total (including overheads)
Hospital 1	£422.23	£509.66	£150.28	£109.33	£0.00	£1191.51	£357.45	£1548.96
Hospital 2	£340.07	£675.37	£81.70	£100.24	£177.33	£1374.72	£412.41	£1787.13
Hospital 3	£284.31	£548.47	£47.77	£100.83	£79.80	£1061.18	£318.35	£1379.53
Hospital 4	£346.34	£643.79	£54.87	£99.25	£177.33	£1321.58	£396.47	£1718.06
Hospital 5	£444.73	£715.48	£73.88	£102.41	£0.00	£1336.51	£400.95	£1737.46
Average	£367.54	£618.56	£81.70	£102.41	£86.89	£1257.10	£377.13	£1634.23

The direct cost of PPV, including both in-theatre and out-of-theatre costs, was £1257.10, which increased to £1634.25 once the 30 % overhead was included.

Reimbursement was at the highest HRG tariff (BZ20Z, £1823 without the MFF uplift, that accounts for unavoidable cost differences of providing healthcare [23]) in 18 cases (32 %), second highest (BZ21Z, £1439) in 34 (60 %) and third highest (BZ22Z, £1013) in 4 (7 %; three of which were coded erroneously as anterior vitrectomy). None were reimbursed under the lowest tariff (BZ23Z, £504) and one phakovitrectomy case was reimbursed erroneously under the cataract tariff (BZ02Z, £704) without consideration of the PPV. The average HRG tariff effectively reimbursed across the 57 cases was £1701.20 including the MFF uplift.

**Figure 1:**
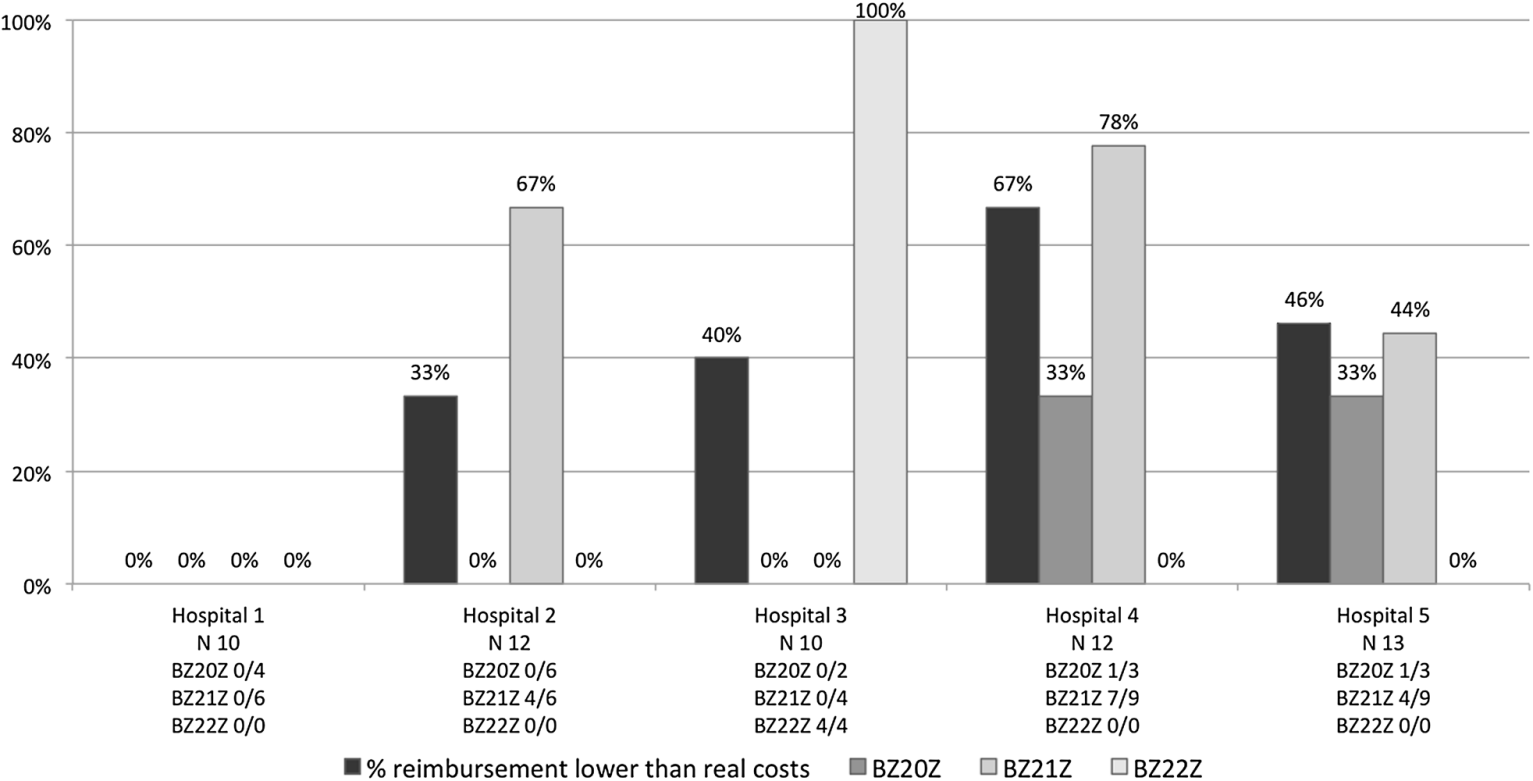
Proportion of cases reimbursed at less than the real costs incurred. The reimbursed cost is compared with the actual costs incurred for all cases and per healthcare resource group (HRG) tariff category; 38.6 % of 57 cases incurred higher costs than their reimbursement. This was 11.1 % of 18 cases in the highest HRG tariff group (BZ20Z), 44.1 % of 34 cases in the second highest group (BZ21Z) and 100 % of 4 cases in the third tariff group (BZ22Z)

When comparing the real costs incurred with the amounts reimbursed, 38.6 % of the 57 cases incurred higher costs than their reimbursement (Fig. 1). This was 11.1 % of cases in the highest HRG tariff group (BZ20Z), 44.1 % of 34 cases in the second highest HRG tariff group (BZ21Z) and 100 % of 4 cases in the third tariff group (BZ22Z). There were moderately large variations in the in-theatre costs across the five hospitals: 39.2 % for staff (range £229.20–£376.70), 68.2 % for equipment (range £47.80–£150.30) and 28.8 % for consumable costs (range £509.70–£715.50).

Consultants advised between 1 and 4 weeks off work depending on the patient’s occupation (mean 2.16 weeks). Post-operative face down head posturing was advised following MH surgery by all consultants, with the advised duration of posturing ranging from 1 to 7 days (mean 3.87 days, median 4 days).

## Discussion

The average cost of performing PPV was estimated to be £1634.25 including overheads (range £1379.55–£1787.15); the average amount reimbursed to the hospitals under the HRG PbR scheme was £1701. Although this finding suggests that the cost incurred by hospitals is close to the reimbursed tariff with a relative difference of 4.1 %, our cost is likely to underestimate the true cost as it did not account for a range of costs incurred outside of the operating room, such as other clinical and non-clinical supplies (e.g. information leaflets) or equipment (e.g. recovery equipment, cardiorespiratory monitor).

The shortfall between hospital costs and reimbursement was most marked for cases coded under the middle tariffs (44.1 % of 34 cases under BZ21Z tariff and 100 % of 4 cases under BZ22Z tariff), suggesting that the reimbursement may be insufficient, or that the codes are incorrectly applied given that cases with peeling should have been coded under BZ21Z and not BZ22Z. Coding was sometimes inconsistent, with the same procedures attracting different reimbursement depending on how the procedure was coded. In addition, one case was erroneously coded as a cataract operation, with no payment for PPV, resulting in a shortfall of £947, and three were erroneously coded as anterior vitrectomy, with a shortfall of £436 each.

There was considerable variation in the discrepancy between cost and reimbursement when comparing different hospitals. One of the main drivers for this difference was based on economies of scale. For example, the higher equipment cost in Hospital 1 can be attributed to the lower number of PPVs performed there, estimated at 250 per year. Other hospitals ranged from 400 and 750 cases annually, which is associated with a lower amortisation cost.

This study did not assess the indirect costs of PPV relating to productivity losses, the burden of surgery, recovery time or lay care. These costs were considered beyond the scope of this study, which aimed primarily to compare the cost-benefit to NHS hospitals, but indirect costs are important when considering the cost-benefit analysis from a patient, health care provider, or societal perspective.

Weaknesses of this study include the fact that the completeness of data collection may have varied across sites, despite standardised training and data collection source documents designed to minimise omissions. Although we deliberately selected a range of representative vitreoretinal units, they may not be representative of all UK vitreoretinal units, and extrapolation to other countries is likely to be of limited use. At present vitreoretinal care is provided by NHS hospitals, but the costs of undertaking vitrectomy may differ if private providers enter the market, although the HRG tariff they will receive from CCG would remain the same. For reasons of commercial sensitivity 1.8 % of item costs were undisclosed and we had to estimate these costs based on equivalent equipment costs disclosed from other units. Out-of-theatre costs were estimated based on accounting costs for patients undergoing PPV for the same conditions, but they were not actually the same patients, and this might introduce error. Likewise, the overhead cost is an estimate, albeit one that is advocated by the Department of Health and widely used.

This study provides data on the major cost drivers influencing the direct cost of performing intermediate complexity PPV in the NHS. It suggests that many hospitals may not be fully reimbursed for PPV, and that the HRG codes and tariffs could be refined to better match hospital costs. Inaccurate coding also contributed to underpayments. It may nonetheless be cost-effective for hospitals to undertake additional PPVs for VMT, ERM and MH, as units undertaking a sufficient volume of work can amortise existing infrastructure. Although this research was set in England, the methodology may provide a useful template for other countries. Further research is needed to estimate the real out-of-theatre costs and indirect costs of PPV.
